# The effects of inhibitory control training for preschoolers on reasoning ability and neural activity

**DOI:** 10.1038/srep14200

**Published:** 2015-09-23

**Authors:** Qian Liu, Xinyi Zhu, Albert Ziegler, Jiannong Shi

**Affiliations:** 1 Institute of Psychology, Chinese Academy of Sciences, Beijing, China; 2 Department of Learning and Philosophy, Aalborg University, Denmark; 3 Department of Educational Psychology, University of Erlangen-Nuremberg, Germany

## Abstract

Inhibitory control (including response inhibition and interference control) develops rapidly during the preschool period and is important for early cognitive development. This study aimed to determine the training and transfer effects on response inhibition in young children. Children in the training group (N = 20; 12 boys, mean age 4.87 ± 0.26 years) played “Fruit Ninja” on a tablet computer for 15 min/day, 4 days/week, for 3 weeks. Children in the active control group (N = 20; 10 boys, mean age 4.88 ± 0.20 years) played a coloring game on a tablet computer for 10 min/day, 1–2 days/week, for 3 weeks. Several cognitive tasks (involving inhibitory control, working memory, and fluid intelligence) were used to evaluate the transfer effects, and electroencephalography (EEG) was performed during a go/no-go task. Progress on the trained game was significant, while performance on a reasoning task (Raven’s Progressive Matrices) revealed a trend-level improvement from pre- to post-test. EEG indicated that the N2 effect of the go/no-go task was enhanced after training for girls. This study is the first to show that pure response inhibition training can potentially improve reasoning ability. Furthermore, gender differences in the training-induced changes in neural activity were found in preschoolers.

Inhibitory control, as a central component of Executive Functions (EFs), involves the ability to inhibit automatic but incorrect responses or to resist interference from distracting stimuli, to reduce a non-target’s impact on ongoing information processing^1^. Response inhibition and interference control are two main types of inhibitory control^1^,^2^,^3^,^4^,^5^. Interference control refers to the ability to prevent the access of distracting information, which has been partially activated (inhibition of attention). In the laboratory, interference control is evaluated using the Stroop task, Simon task, and flanker task. Response inhibition refers to the suppression of a pre-potent or automatic response (inhibition of action). It is assessed by the go/no-go task, continuous performance task (CPT), and stop-signal task in the laboratory^4^,^5^,^6^. Inhibitory control plays an important role in early cognitive development^7^,^8^,^9^; meanwhile, it can predict school readiness for preschoolers and school achievement in mathematics and reading^10^,^11^,^12^.

Inhibitory control develops quickly in early childhood, particularly between 3 and 6 years of age^13^,^14^,^15^. The development of inhibitory control depends on the functional maturation of the frontal system. The rapid growth of inhibitory control is assumed to reflect changes in the prefrontal cortex (PFC) during the preschool years^16^,^17^. Event-related potentials (ERPs) can be used as a sensitive measure of the developmental difference between response inhibition and interference control. The difference waveform of the N2 component associated with response inhibition was maximal at central scalp sites in children, but was maximal at frontal sites in adults, while the incongruent N2 effect for interference control may not be observed in young children (8–11 years old)^4^. Compared to interference control, response inhibition develops earlier and plays a more fundamental role in early cognitive development. In previous ERP studies using go/no-go tasks, larger amplitudes of the N2 (200–300 ms) component have been found for successful responses to no-go trials compared to go trials, which reflects response inhibition in adults^13^,^18^,^19^. However, the N2 component of young children is usually observed between 250 and 500 ms after stimulus onset^20^,^21^, and the no-go N2 effect is larger and more widely distributed across the fronto-parietal electrodes^20^,^21^,^22^. Considering the importance of inhibitory control and neural plasticity during early childhood^14^,^17^,^23^,^24^,^25^, we focused on inhibitory control training, especially response inhibition training for preschoolers in the present work.

Increased attention has been devoted to investigating inhibitory control training in children from different age groups. Dowsett and Livesey performed a study in which they trained children (3–5 years old, 15–20 min/day for 3 days) from low socio-economic-status families^26^. In their study, 16 children were trained using a manual go/no-go task and 16 children were trained using the Wisconsin Card Sorting Test and stop-signal task. The results showed that the performance of both groups on the go/no-go task improved. Thorell and colleagues found that inhibitory control training (go/no-go task, stop-signal task, and flanker task) in preschoolers (4–5 years old, 15 min/day for 5 weeks) only improved the performance on the training tasks themselves^27^. Rueda and colleagues trained children (4- and 6-year-olds, 5 training days in total over 2–3 weeks) using three tasks (including a Stroop-like task); however, while there were no group differences in the behavioral data except for performance on the Kaufman Brief Intelligence Test, the trained children showed “adult-like” brain activity (more efficient and faster activation) in response to the flanker task^28^. Subsequent work replicated their results and showed that some (weaker) of the training-induced changes lasted for 2 months^29^. There may be several reasons for the inconsistent results found in these studies. First, EEG recordings are more sensitive than behavioral assessments, thus the training-induced changes may be more noticeable in the EEG data. Second, to maintain the interest of young children, various tasks (not just those involving inhibitory control) were combined as training tasks for preschoolers^27^,^28^. However, the relative heterogeneousness of such training tasks makes it impossible to determine which training task is responsible for inducing the training effects or which cognitive component is being trained. Third, it should be noted that the different forms of inhibitory control (response inhibition and interference control), which were combined as training tasks in Thorell’s research, may vary in how easily they can be improved through training. Therefore, using a single training task with a more pure cognitive component and in which the specific type of inhibitory control is clearly established is a better choice. In addition, the training task should be child-friendly, interesting, and challenging. Meanwhile, touch-screen technology and motion-contingent interfaces can be employed for young children to provide a more vivid training environment.

Furthermore, conducting a cognitive training experiment can improve our understanding of the causal interactions between the trained and related non-trained cognitive constructs, which can ameliorate the disadvantages of correlational studies that only focus on early cognitive development^30^. Consequently, researchers treasure the near transfer effect (improvement on non-trained tasks measuring the same cognitive component) and far transfer effect (improvement on non-trained tasks measuring related cognitive components) more than they do within-task training improvement^31^. Currently, inhibitory control is usually involved in the transfer tasks employed in cognitive training studies^32^,^33^,^34^,^35^. In the relatively few inhibitory control training studies that have been performed on young children, transfer effects were not easily found. Although previous studies have demonstrated a close relationship between inhibitory control and fluid intelligence^36^,^37^,^38^,^39^, it remains unclear whether inhibitory control training can be transferred to fluid intelligence, as limited research exits on this topic. Reasoning ability (one main aspect of fluid intelligence) was facilitated in a cognitive training program for 5-year-old children^29^. However, it was difficult to determine whether the improvement was due to inhibitory control training (a Stroop-like task), since two other cognitive components were also trained. Working memory (another key EF component^1^) was the focus of another inhibitory control training study for preschoolers, but no transfer effect on working memory was found^27^. The training duration for each task (one for interference control and two for response inhibition) may have been too limited to induce transfer effects on working memory. Therefore, it is necessary to train one type of inhibitory control directly, namely response inhibition, in order to investigate its transfer effects on interference control, working memory, and fluid intelligence in preschoolers.

The new trend in cognitive training research is to investigate the training-induced neural changes rather than just focusing on the behavioral changes. For inhibitory control training, Schapkin and colleagues found an increase in the frontal no-go N2 for adults after three daily training sessions using the go/no-go task^40^. In another study on healthy adults, Manuel *et al.* found a significant reduction in left parietal activity to no-go stimuli following 30 min of practice on the go/no-go task^41^. A marginal significant increase in the frontal no-go N2 was identified after computer-based working memory and response inhibition training for children with attention deficit/hyperactivity disorder^42^. Using these training programs, researchers demonstrated a training-related strengthening of the inhibition mechanism by recruiting individuals after middle childhood as participants. Rueda and colleagues enrolled 4-year-old and 6-year-old children and found training-induced changes at the neural level, but not at the behavioral level, when using the flanker task^28^,^29^. Until now, no evidence existed regarding the neural changes induced by response inhibition training in young children. Considering the aforementioned developmental differences between the two types of inhibitory control, response inhibition training studies that focus on the neural changes in early childhood are needed. As mentioned above, ERP data can provide more sensitive and specified information than behavioral data can, since behavioral data merely reflect the overt outcome of covert information processing. Furthermore, EEG recordings can investigate the actual brain processes employed during task performance, thus revealing the subtle physiological effects that result from training, possibly before overt effects become apparent.

Another new direction in cognitive training is to focus on individual differences in predicting training-related transfer effects and brain changes. Researchers highlight that it is important to consider whether some individuals benefit more from training than others^30^,^43^,^44^. As a dimension of individual difference, gender was often ignored or controlled for in previous inhibitory control training programs. The lack of evidence regarding gender differences in training-induced gains provides a new perspective for our response inhibition training program. Therefore, the aim of this study was to investigate the training and transfer effects of response inhibition in 4-year-old children by collecting both behavioral and electrophysiological data. We hypothesized that response inhibition training would: (1) improve participants’ performance on the training task; (2) promote participants’ performance on the non-trained task, which reflected response inhibition or related cognitive constructs; (3) lead to changes in brain activity, as indicated by the N2 effect; and (4) show gender differences in the gains of behavior and neural activity.

## Methods

### Participants

All participants were recruited from a community kindergarten. They were randomly allocated into a training group (N = 20; 12 boys, mean age: 4.87 ± 0.26 years) or a control group (N = 20; 10 boys, mean age: 4.88 ± 0.20 years). The two groups were comparable in terms of their parents’ educational background. Informed consent was obtained from all parents. According to the parents’ questionnaire, all participants (right handed, full-term birth, normal or corrected-to-normal vision) were free from neurological or psychiatric disorders and had not used a tablet computer before. All experiments were performed in accordance with the guidelines and were approved by Institutional Review Board for Institute of Psychology.

### Training session

The specific cognitive component targeted by the training program was response inhibition, which is extensively assessed by the go/no-go, CPT, and stop-signal paradigm. An iPad tablet computer (Apple Inc., CA, USA) was used as a training device. The entirely new, interesting training task was the classic mode of “Fruit Ninja” (a commercial game, Halfbrick Studios, Brisbane, Australia), which involves an individual’s cognitive ability of response inhibition. There were two types of stimuli (fruit or a bomb), which were presented randomly. Participants were instructed to respond only to fruits (go stimuli), and to inhibit their responses to bombs (no-go stimuli). Participants were asked to slice any of the fruits (go), but to avoid touching the bombs (no-go). When a fruit was sliced, it was recorded as one positive point. The game was over when a bomb was touched or three fruits were missed, which was counted as a run. The score of each run was recorded, and the final score of “Fruit Ninja” is the mean value of the three best scores. A training goal was set for the participants to provide them with a challenge, such as “Please slice thirty more fruits than you did last time.” Participants played the game for 15 min on each training day. All individuals in the training group had 4 training days per week for 3 weeks (12 sessions in total). The experimenter ensured that participants stayed on task and that they were motivated by providing encouragement and setting goals according to their individual performance. Participants in the active control group played an irrelevant coloring game on the iPad tablet computer (15 min per day, twice a week for 3 weeks) that required similar motor responses, but was less cognitively demanding. During each session, participants could choose to color two figures they liked and then rested between the figures. Children were praised for painting the figures with more colors and for naming the colors.

### Pre-test and post-test tasks

All children were individually tested in a quiet room at their kindergarten. The duration of the test was about 17–25 min per person (excluding EEG recording for the go/no-go task). They underwent tests on interference control (see the section “the adapted version of the day-night Stroop task” for details), which lasted for 3 min^27^,^45^; the digit span subtests of the Wechsler Preschool and Primary Scale of Intelligence-III (WPPSI-III) for 5–8 min, and nonverbal abstract reasoning ability test (Raven’s Colored Progressive Matrices Test, 36 items) for 9–14 min. EEG recording for the go/no-go task was completed in a dimly lit and electrically shielded booth (see sections “The go/no-go task” and “EEG recording” for details). Three graduate students majoring in psychology collected the experimental data.

#### The adapted version of the day-night Stroop Task

Participants were instructed to tell the experimenter the opposite as fast as possible when a certain image was presented randomly by card. Thus, children were required to inhibit a dominant response (e.g., saying “day” when shown a drawing of the sun, saying “girl” when shown a drawing of a girl,) when faced with a salient conflict in order to produce a non-dominant response (e.g., saying “day” when shown a drawing of a moon, saying “girl” when shown a drawing of a boy). There were 32 cards in total and each card lasted for 2 s. Ten practice cards were administered before the test. There was no feedback for the test. The number of correct answers given the first time was used as the outcome measure.

#### The go/no-go task

The stimuli in this response inhibition task were two pairs of pictures (moon and star, car and helicopter). Stimuli were presented in the center of the screen with a visual angle of 5° vertically and horizontally. Stimuli were presented for 200 ms with a random inter-stimulus interval of 1100–1400 ms. During each trial of the first block, participants were instructed to respond (press a button on the keyboard) when the moon was presented (go trial) and not to respond when the star was presented (no-go trial). After an initial practice block of 40 stimuli, two experimental blocks, each consisting of 100 stimuli (40% no-go probability), were completed with 1–2 min breaks between blocks. Stimulus presentation and behavioral data acquisition were performed by the E-prime software system (PST Inc., Pittsburgh, PA, USA). Reaction time and the number of correct responses were recorded. For the go response, half of the participants were instructed to press the space bar with the forefinger of their left hand, while the other half of the participants was instructed to use the forefinger of their right hand. The number of false alarms (i.e., making a response when instructed not to respond) in the go/no-go task was used to measure response inhibition.

#### EEG recordings

EEG data (32-channel, amplified by SynAmps 2 online, bandwidth: 0.05–200 Hz, sampling rate: 1000 Hz) were recorded with Ag-AgCl electrodes according to the International 10–20 placement system (Neuroscan Inc., USA). All sites were referenced to the left mastoid online. The vertical and horizontal electrooculograms were recorded with two pairs of electrodes, one placed above and below the left eye, and another was placed 10 mm from the outer canthi of both eyes. Electrode impedances were kept below 8 kΩ. Segmented files were scanned for eye and movement artifacts. EEG epochs of 800 ms, including 100 ms of pre-stimulus time as baseline, were averaged offline using correct trials according to the stimuli (go, no-go). Epochs with artifacts exceeding ±100 μV at any electrode were omitted from further analysis. During analysis, the data were band-pass filtered offline (0.05 to 30 Hz) and recomputed to average the reference.

## Results

All statistical analyses were conducted using SPSS 16.0 (IBM Inc., NY, USA). Behavioral data from four children with scores two standard deviations below the mean in the Raven (N = 1) and Digit span (N = 3) pretests were excluded from analysis. Thus, 36 children completed the behavioral study, with 16 in the training group (9 boys, mean age: 4.92 ± 0.27 years) and 20 in the control group (10 boys, mean age: 4.85 ± 0.21 years). In addition, three children refused to complete the EEG recordings in the post-session and another three children dropped out of the study. Therefore, their EEG data were also excluded from analysis. Thus, 34 children completed the EEG study, with 19 in the training group (11 boys, mean age: 4.90 ± 0.28 years) and 15 in the control group (6 boys, mean age: 4.87 ± 0.23 years). The groups did not differ on any of the behavioral measures collected at pre-test, as examined by one-way analyses of variance (ANOVAs; ps > .113, partial η^2^ < .072, two-tailed for all comparisons).

### Analysis of the performance on the training task

The “Fruit Ninja” game scores for both groups were recorded on the first and last training days. In order to study the participants’ improvement over time on the training task “Fruit Ninja”, we ran a 2 (group: training vs. control) × 2 (gender: boy vs. girl) analysis of covariance (ANCOVA; two-tailed) with the scores of the post-test performance as the dependent variable and the scores of the pre-test performance as the covariate. The results revealed a significant main effect of group (F_(1, 35)_ = 216.994, p < .001, partial η^2^ = .861) but no effect of gender (F_(1, 35)_ = .879, p = .355, partial η^2^ = .025). The post-test performance of the training group (102.45 ± 15.00) was significantly better than that of the control group (57.05 ± 13.48) after controlling for the pre-test performance of the training (46.15 ± 13.13) and control (51.90 ± 15.11) groups. The interaction effect between gender and group (F_(1, 35)_ = 1.624, p = .211, partial η^2^ = .044) was not significant, indicating that the performance difference on “Fruit Ninja” between the two groups after training was not influenced by gender.

### Analysis of the behavioral data for the non-trained tasks

The means and standard deviations of the pre- and post-test scores during the non-trained tasks for both groups are presented in Table 1. In order to investigate the transfer effect, we ran a 2 (group: training vs. control) × 2 (gender: boy vs. girl) ANCOVA (two-tailed) with the scores of the post-test performance as the dependent variable and the pre-test performance scores as the covariate in each non-trained task (Bonferroni corrected for six comparisons, threshold at 0.05/6 = 0.008). For the go/no-go task, no significant effects of the group (F_(1, 29)_ = .125, p = .727, partial η^2^ = .004) or gender (F_(1, 29)_ = 1.046, p = .315, partial η^2^ = .035) were observed. The interaction effect between gender and group (F_(1, 29)_ = 3.303, p = .080, partial η^2^ = .102) was not significant, revealing that the participants’ performance on the go/no-go task did not change after training. For the Stroop task, the main effects of group (F_(1, 31)_ = 1.681, p = .204, partial η^2^ = .051) and gender (F_(1, 31)_ = 1.033, p = .317, partial η^2^ = .032), and the group by gender interaction (F_(1, 31)_ = .418, p = .523, partial η^2^ = .013) all failed to reach significance, indicating that the participants’ performance on the Stroop task did not change after training. Regarding the participants’ performance on the Raven’s test, the main effect of group showed a considerable trend towards significance (F_(1, 31)_ = 4.962, p = .033, partial η^2^ = .138), but there was no effect of gender (F_(1, 31)_ = .436, p = .514, partial η^2^ = .014). The gender by group interaction effect did not reach significance (F_(1, 31)_ = 3.505, p = .071, partial η^2^ = .102), suggesting that the differences in performance on the Raven’s test between the two groups after training did not differ between the two genders. The performance index for the digit span subtest of the WPPSI-III was the mean value of points on both the forward and backward conditions. Results indicated that the main effects of group (F_(1, 31)_ = .774, p = .386, partial η^2^ = .024) and gender (F_(1, 31)_ = .026, p = .872, partial η^2^ = .001), and the group by gender interaction (F_(1, 31)_ = .285, p = .597, partial η^2^ = .009) were not significant, suggesting that the participants’ performance on working memory tasks did not change after response inhibition training.

### Analysis of the EEG data for the go/no-go task

**Figure 1:**
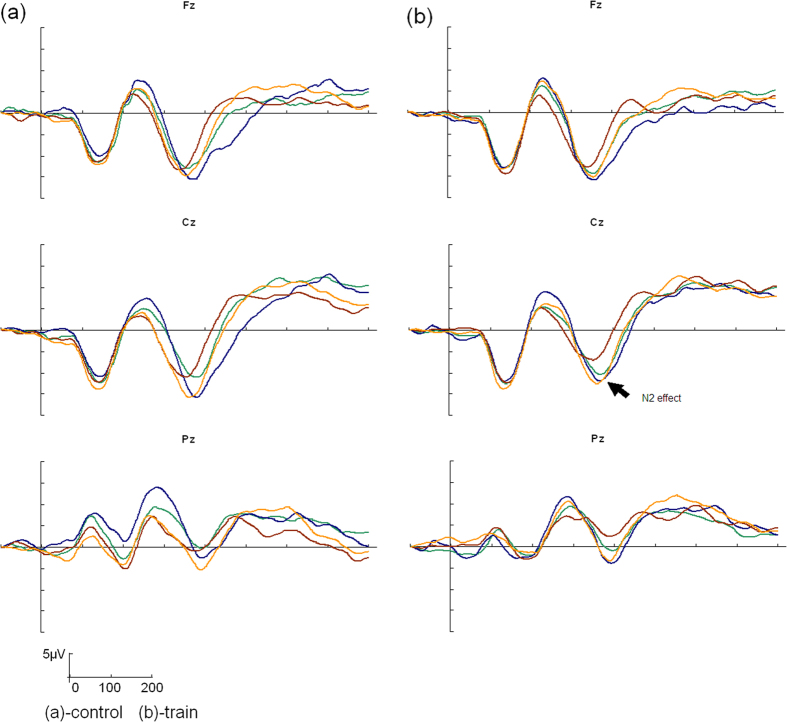
Grand mean ERPs over three midline channels during go and no-go trials of the go/no-go task in pre-test (go trials, green; no-go trials, blue) and post-test (go trials, red; no-go trials, yellow) for each group.

The N2 effect of response inhibition for young children is most obvious in the central areas and is more posterior than it is in adults^22^,^46^. Three midline sites were used for analysis^47^: Fz, Cz, and Pz. N2 mean amplitudes (time window 320 to 400 ms) were analyzed using mixed model ANOVAs (two-tailed) with session (pre-test vs. post-test) × stimulus (go trials vs. no-go trials) as within-subject factors and group (training vs. active control) as a between-subjects factor. Post hoc pair-wise comparisons with Bonferroni adjustments were carried out for all significant interactions. Mixed model ANOVAs of the N2 amplitude at electrodes Fz, Cz, and Pz revealed no significant three-way interactions or two-way interactions (ps > .196, partial η^2^ < .052, two-tailed for all comparisons). A main effect of stimulus type was found at Fz, Cz, and Pz (see Table 2), indicating that the traditional no-go > go effect for N2 was replicated. Grand mean ERPs over these three midline channels was shown in Fig. 1. We measured the N2 effect in the no-go minus go difference waveforms^19^,^48^,^49^. In order to study the change in the N2 effect on the go/no-go task, we ran a 2 (group: training vs. control) × 2 (gender: boy vs. girl) ANCOVA (two-tailed) with the post-test N2 effect as the dependent variable and pre-test N2 effect as the covariate at each electrode. No effect of group (F_(1,29)_ = 1.269, p = .269, partial η^2^ = .042) was found, but a main effect of gender (F_(1, 29)_ = 7.538, p = .010, partial η^2^ = .206) and a significant interaction effect between gender and group were found at the Cz electrode (F_(1, 29)_ = 4.974, p = .034, partial η^2^ = .146). The simple effect analyses revealed that the N2 effect was larger in the training group than it was in the control group for girls (F_(1, 14)_ = 5.01, p = .032), whereas for boys, no effect of group was observed (F_(1, 14)_ = 2.39, p = .133). The simple effects of gender within the groups showed that the N2 effect in girls was larger than that in boys only within the training group (F_(1,14)_ = 15.34, p < .000). No significant main effects of gender (F_(1, 29)_ = 2.411, p = .131, partial η^2^ = .077) or group (F_(1,29)_ = .226, p = .638, partial η^2^ = .008) or gender by group (F_(1, 29)_ = .105, p = .748, partial η^2^ = .004) were observed at the Fz electrode. For the Pz electrode, there were also no significant effect of gender (F_(1, 29)_ = .731, p = .400, partial η^2^ = .025), group (F_(1,29)_ = .137, p = .714, partial η^2^ = .005), or a gender by group interaction (F_(1,29)_ = 3.111, p = .088, partial η^2^ = .097). Table 3 displays the means and standard deviations for the pre- and post-test N2 effects in both groups during the go/no-go task.

## Discussion

This study is the first to focus on response inhibition training in children below school-age. We assessed pre- and post-training performance while collecting ERPs during a go/no-go task and several important findings emerged. First, participants in the training group improved their performance on the training task (the game of “Fruit Ninja”), which demonstrated that the efficiency of response inhibition can be improved with training for preschoolers. If the training task were too easy or too difficult, the children’s performance on the training task would hardly improve. Therefore, the training effect that we observed also illustrates that the difficulty level of the training task was appropriate for the young children. Second, the near transfer effect of response inhibition training, which is more important, was examined during the go/no-go task. However, the lack of a near transfer effect was somewhat unexpected. Theoretically, transfer may occur if both the training and transfer tasks share a common cognitive mechanism and activate similar brain regions or networks. The training task, “Fruit Ninja”, and the near transfer task, “go/no-go task,” both require the participants to make a response to certain stimuli (go condition), while inhibiting their response to other competing stimuli (no-go condition). The efficiency of the neural pathway underlying response inhibition was improved through training and, in theory, should transfer to the performance of the go/no-go task. The absence of a near transfer effect may be due to the relative low sensitivity of behavioral data. However, the results are in line with an interference control training study for preschoolers. Training-induced changes at the neural level, but not at the behavioral level, were found in the near transfer task (the flanker task)^28^. Therefore, we also examined whether response inhibition training would induce changes at the neural level.

We demonstrated that behavioral training induced brain activity changes, but only in girls, as indexed by a significant increase in the midline central N2 effect of the go/no-go task for girls in the training group compared to the control group. This effect of cognitive training was similar to the influence of individual maturation^21^,^22^,^50^. Therefore, the development of response inhibition may be facilitated after training in preschool-age girls. Several studies involving inhibitory control training also reported brain activity changes. However, almost all of them recruited adult participants^25^,^41^,^51^,^52^,^53^. Inhibitory control training for preschoolers currently lacks experimental evidence from a cognitive neuroscience perspective. We showed that playing “Fruit Ninja” can differentially enhance the N2 effect of girls and boys that is related to the go/no-go task; specifically, the level of neural activity for girls is easier to improve with training. It is worth noting that the main effect of group was not significant, as the training-induced neural changes for girls were masked by the absence of changes for boys. This finding implies that the underlying neural plasticity for girls is different from that for boys, which has been overlooked in the majority of previous cognitive training studies.

Previous studies have demonstrated that certain kinds of cognitive training (e.g., working memory and executive attention) can influence fluid intelligence^28^,^32^,^54^,^55^. However, it is unclear whether response inhibition training can be transferred to fluid intelligence. To some extent, our results imply that response inhibition training may show a trend to be transferred to reasoning. Although the improvement on Raven’s Progressive Matrices observed in our study was not significant after correcting for multiple comparisons, the effect size still showed that response inhibition training is a promising way to improve reasoning ability. Reasoning ability heavily depends on inhibitory control, i.e., blocking information irrelevant to the target^9^,^36^. The training program may facilitate preschoolers’ ability to inhibit inappropriate responses and ignore irrelevant environmental distractions. Accordingly, in the future, we will recruit more participants to examine whether there is stable significant progress on reasoning ability. Prior to our study, it was unclear whether response inhibition training could be transferred to reasoning abilities. Other researchers adopted heterogeneous training tasks (inhibitory control and two other cognitive components) in a previous training program for preschoolers and found that their reasoning abilities were facilitated^28^,^29^. However, it is difficult to determine whether the improvement was due to inhibitory control training. Here, for the first time, we showed the potential transfer effect on reasoning using a pure response inhibition training task.

The lack of a transfer effect on working memory was revealed in an inhibitory control training study^27^, in which interference control and response inhibition were both trained. We used only one response inhibition training task and still did not find a transfer effect on working memory. This result was possibly due to the developmental fractionation of working memory and response inhibition at preschool age. The relationship between these two key EF components was found to change developmentally in a correlational study, showing that these components begin to become independent from each other after 4 years of age^8^. This change reflects the functional differentiation of an individual’s PFC with development^17^. Brain activation increases in the ventromedial PFC during response inhibition^13^,^56^, while in the dorsolateral PFC during working memory^57^,^58^,^59^. Response inhibition training activates the specific area of the cortex that underlies response inhibition, and appears not to influence working memory. The lack of a transfer effect on the Stroop task illustrated that the training of response inhibition does not transfer to interference control. It has previously been demonstrated that training using the Stroop task does not transfer to the go/no-go task for the elderly^60^. A possible explanation is that response inhibition and interference control are two fundamentally distinct types of inhibitory control, and that the differences between them make the transfer impossible^1^,^2^,^4^,^6^. Nevertheless, we need to be cautious not to overgeneralize our results. The lack of transfer effects on the Stroop task may be linked to our presentation method. When preparing for our experiment, we found that children in preschool were not familiar with computerized tasks. Therefore, only the go/no-go task was presented using a computer, while the other tasks were presented using traditional paper and pencil methods. Therefore, the indicator of reaction time, which is more sensitive, could not be measured. Additionally, we only recorded behavioral data for the Stroop task. Perhaps significant transfer effects may be demonstrated when EEG data is included.

### Implications and limitations

We believe that the present study makes a novel contribution to the literature by providing evidence that pure response inhibition training for preschoolers can improve their task performance and potentially enhance their reasoning abilities. Moreover, this study is the first to demonstrate a gender difference in the training-induced changes in the ERP component related to response inhibition in early childhood. We recorded brain activity during the non-trained task (go/no-go), which provided new information on the possible underlying neural mechanisms responsible for inhibition and its plasticity in the developing brain at an early age. These findings hold implications for developing effective inhibitory control training programs for preschoolers.

However, several limitations exist in the present study. First, although we organized an active control group, which is better than a passive control group, the amount of training in the active control group (10 min/day with a 5 min rest interval, 1–2 days/week for 3 weeks) was not equal to that in the training group (15 min/day, 4 days/week for 3 weeks). Thus, the improvements in performance and changes in brain activity in the training group may be partially explained by the differences in the amount of training between the two groups. We acknowledge this major limitation and believe that the effect of response inhibition training observed in this study will remain after controlling for this disadvantage in our future research. Second, the dropout rate of the control group was higher than that of the training group (5% vs. 25%) for the ERP data; meanwhile, the dropout rate of the control group was lower than that of the training group for the behavioral data (20% vs. 0%). Although the data from these participants were excluded from the analyses, the small sample size did harm the explanatory power of our results. In future studies, a larger sample size will be adopted so that the dropout rates of the two groups can be controlled. Third, the participants’ scores on “Fruit Ninja” were only recorded on the first and last training days. If we record the participants’ performance for all 12 training sessions, a line graph indicating the specific changes could be shown and afford additional information. Additionally, we only examined the post-test period immediately after training, but it is worth checking the participants’ performances 2 months after training. Future studies may focus on the duration of these effects.

## Additional Information

**How to cite this article**: Liu, Q. *et al.* The effects of inhibitory control training for preschoolers on reasoning ability and neural activity. *Sci. Rep.* **5**, 14200; doi: 10.1038/srep14200 (2015).

## Figures and Tables

**Table 1:** Pre- and post-test performances on the non-trained tasks for the two groups.

Task		**Training group**		**Active control group**	
		Pre-testMean (SD)	Post-testMean (SD)	Pre-testMean (SD)	Post-testMean (SD)
Digit span forward	boys	7.6 (1.4)	7.8 (1.7)	7.6 (1.3)	7.4 (1.3)
	girls	7.9 (1.1)	7.7 (0.8)	7.0 (1.9)	7.1 (1.7)
Digit span backward	boys	3.9 (1.7)	3.8 (1.6)	2.9 (0.7)	2.8 (0.4)
	girls	2.3 (0.5)	2.6 (0.5)	2.3 (0.7)	2.4 (0.5)
Advanced Stroop	boys	28.2 (1.6)	28 (1.7)	27.7 (1.9)	26.8 (2.1)
	girls	27.9 (1.5)	28 (0.8)	26.9 (2.2)	27.2 (1.5)
Raven’s Matrices	boys	22.4 (5.2)	26.4 (4.8)	21.2 (4.7)	20.2 (6.8)
	girls	18.6 (1.8)	20.4 (2.9)	18.2 (3.3)	19.7 (2.5)
False Alarm (%)	boys	22.4 (18.2)	15.7 (14.5)	15.8 (5.3)	14.8 (5.9)
	girls	17.5 (15.9)	17.5 (14.2)	16.2 (12.1)	13.6 (13.0)

Digit span forward test and Digit span backward test are from the WPPSI-III (the Wechsler Preschool and Primary Scale of Intelligence-III), False Alarm is the percentage of no-go trials participants pressed.

**Table 2:** Main effect of stimulus type at the FZ, CZ, and PZ electrodes for the go/no-go task.

	No-go Mean(SD)	Go Mean(SD)	**Main Effect**	Partialη^2^
FZ	−13.1 (4.3)	−10.9 (5.2)	P = .019*	.161
CZ	−11.3 (5.4)	−7.8 (5.0)	P = .000***	.333
PZ	0.6 (7.1)	2.6 (5.4)	P = .034*	.133

*< .05,***< .001.

**Table 3:** Pre- and post-test N2 effect for the go/no-go task in the two groups at the FZ, CZ, and PZ electrodes.

		**Training (N = 19)**		**Active control (N = 15)**	
		Pre-test Mean(SD)	Post-test Mean(SD)	Pre-test Mean(SD)	Post-test Mean(SD)
FZ	boys	−0.1 (7.1)	−0.4 (7.2)	−3.7 (5.2)	−0.8 (10.2)
	girls	−4.4 (4.1)	−6.4 (5.7)	−0.5 (4.4)	−3.5 (8.0)
CZ	boys	−0.2 (8.7)	−1.4 (3.6)	−4.6 (8.1)	−4.2 (4.3)
	girls	−3.2 (4.0)	−11.0 (6.8)	−1.6 (3.9)	−4.5 (5.9)
PZ	boys	−0.5 (8.7)	−1.7 (5.6)	−0.8 (5.7)	−3.4 (4.4)
	girls	−3.4 (4.1)	−6.8 (10)	−0.8 (5.2)	−0.7 (2.0)

The N2 effect was computed as the no-go minus go difference waveforms.
