# Corrigendum: The effects of inhibitory control training for preschoolers on reasoning ability and neural activity

**DOI:** 10.1038/srep20296

**Published:** 2016-02-04

**Authors:** Qian Liu, Xinyi Zhu, Albert Ziegler, Jiannong Shi

Scientific Reports 5: Article number: 1420010.1038/srep14200; published online: 09232015; updated: 02042016

In this Article, an additional affiliation for Qian Liu and Xinyi Zhu was omitted. The correct affiliation is listed below:

University of Chinese Academy of Sciences, Beijing 100101, China

